# Unlocking the potential of co-applied biochar and plant growth-promoting rhizobacteria (PGPR) for sustainable agriculture under stress conditions

**DOI:** 10.1186/s40538-022-00327-x

**Published:** 2022-08-22

**Authors:** Laraib Malik, Muhammad Sanaullah, Faisal Mahmood, Sabir Hussain, Muhammad Hussnain Siddique, Faiza Anwar, Tanvir Shahzad

**Affiliations:** 1 Department of Environmental Sciences, Government College University Faisalabad, Allama Iqbal Road, Faisalabad, 38000 Pakistan; 2 Institute of Soil and Environmental Sciences, University of Agriculture, Faisalabad, Pakistan; 3 Department of Bioinformatics and Biotechnology, Government College University Faisalabad, Allama Iqbal road, Faisalabad, Pakistan

**Keywords:** Soil amendments, Soil quality, Crop growth, Biochar, PGPR, Drought stress, Salinity stress

## Abstract

Sustainable food security is a major challenge in today’s world, particularly in developing countries. Among many factors, environmental stressors, i.e., drought, salinity and heavy metals are major impediments in achieving sustainable food security. This calls for finding environment-friendly and cheap solutions to address these stressors. Plant growth-promoting rhizobacteria (PGPR) have long been established as an environment-friendly means to enhance agricultural productivity in normal and stressed soils and are being applied at field scale. Similarly, pyrolyzing agro-wastes into biochar with the aim to amend soils is being proposed as a cheap additive for enhancement of soil quality and crop productivity. Many pot and some field-scale experiments have confirmed the potential of biochar for sustainable increase in agricultural productivity. Recently, many studies have combined the PGPR and biochar for improving soil quality and agricultural productivity, under normal and stressed conditions, with the assumption that both of these additives complement each other. Most of these studies have reported a significant increase in agricultural productivity in co-applied treatments than sole application of PGPR or biochar. This review presents synthesis of these studies in addition to providing insights into the mechanistic basis of the interaction of the PGPR and biochar. Moreover, this review highlights the future perspectives of the research in order to realize the potential of co-application of the PGPR and biochar at field scale.

**Graphical Abstract:**

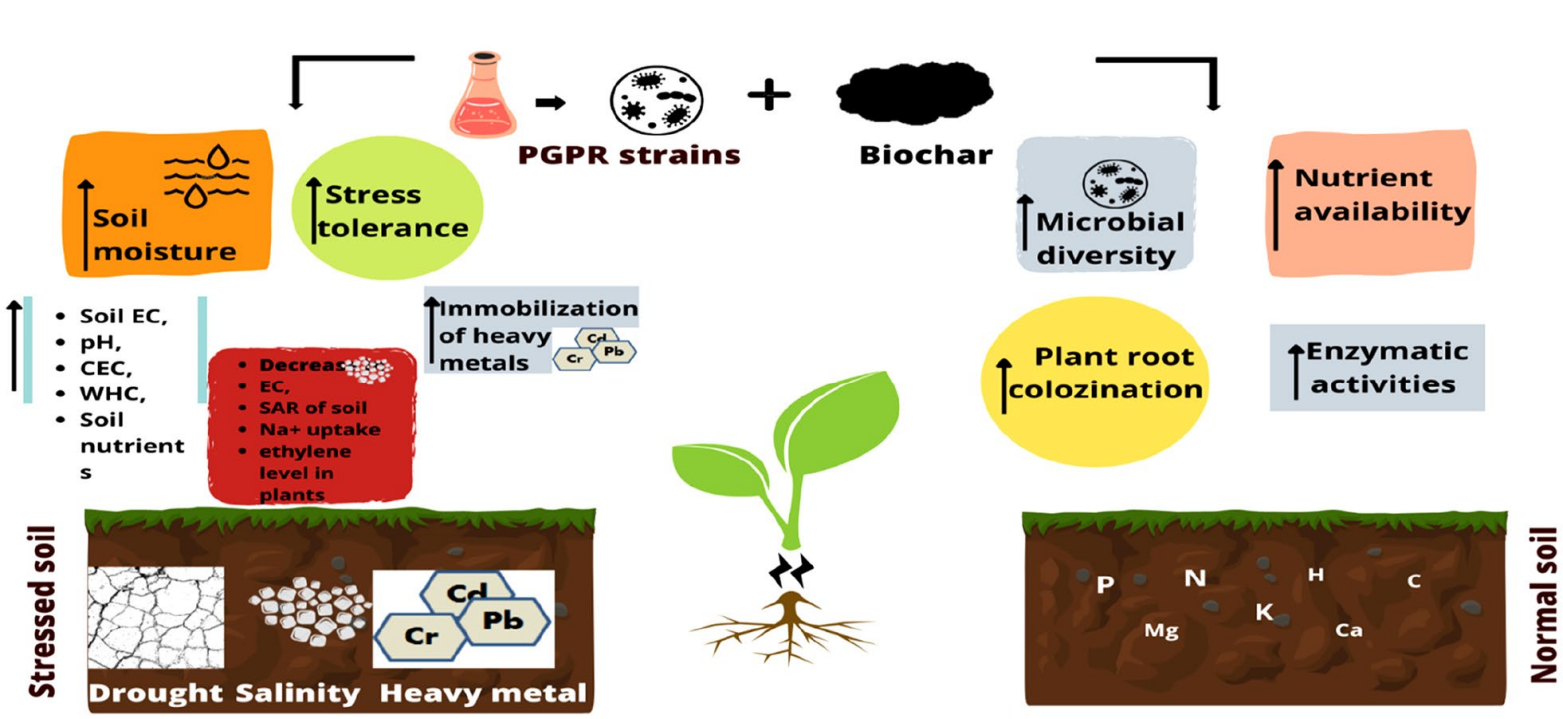

## Introduction

Recently in agro-ecosystems, soil amendments are used to support plant growth and development, especially by adding organic and inorganic nutrients to the soil. Soil amendments are elements that are added to the soil to improve its ability to support plant life [1]. Soil amendments such as compost, animal slurry, savage sludge, green manure, farm yard manure, fly ash, biochar (BC), PGPR (plant growth-promoting rhizobacteria), etc., are the organic soil amendments have been explored as innovative strategies to increase crop productivity and soil fertility [2–6]. Numerous previous studies have shown that soil organic amendments can provide various benefits to soil such as improved soil texture, increased soil fertility, long-term maintenance of soil health, and in particular, crop yields [7–9].

However, the application of organic soil amendments to agricultural soils poses a number of threats to the agro-ecosystem and human health. Organic soil amendments often include a range of pollutants, including heavy metals, potential human pathogens, persistent organic pollutants, and emerging pollutants. From the emerging pollutants the presence of antibiotic-resistant bacteria, antibiotic residues, and antibiotic-resistant genes in agricultural organic amendments is of great concern at the moment, due to the harmonious risks to human health [10]. Soil amendments should have characteristic such as environmental protection and should not have a negative impact on soil structure, soil fertility, or the ecosystem as a whole [11]. PGPR and biochar due to their different properties has attracted growing interest in the last few years to be the promising soil amendments in reducing risk associated with other soil amendments application under normal and stressed conditions [4, 12–16].

**Fig. 1:**
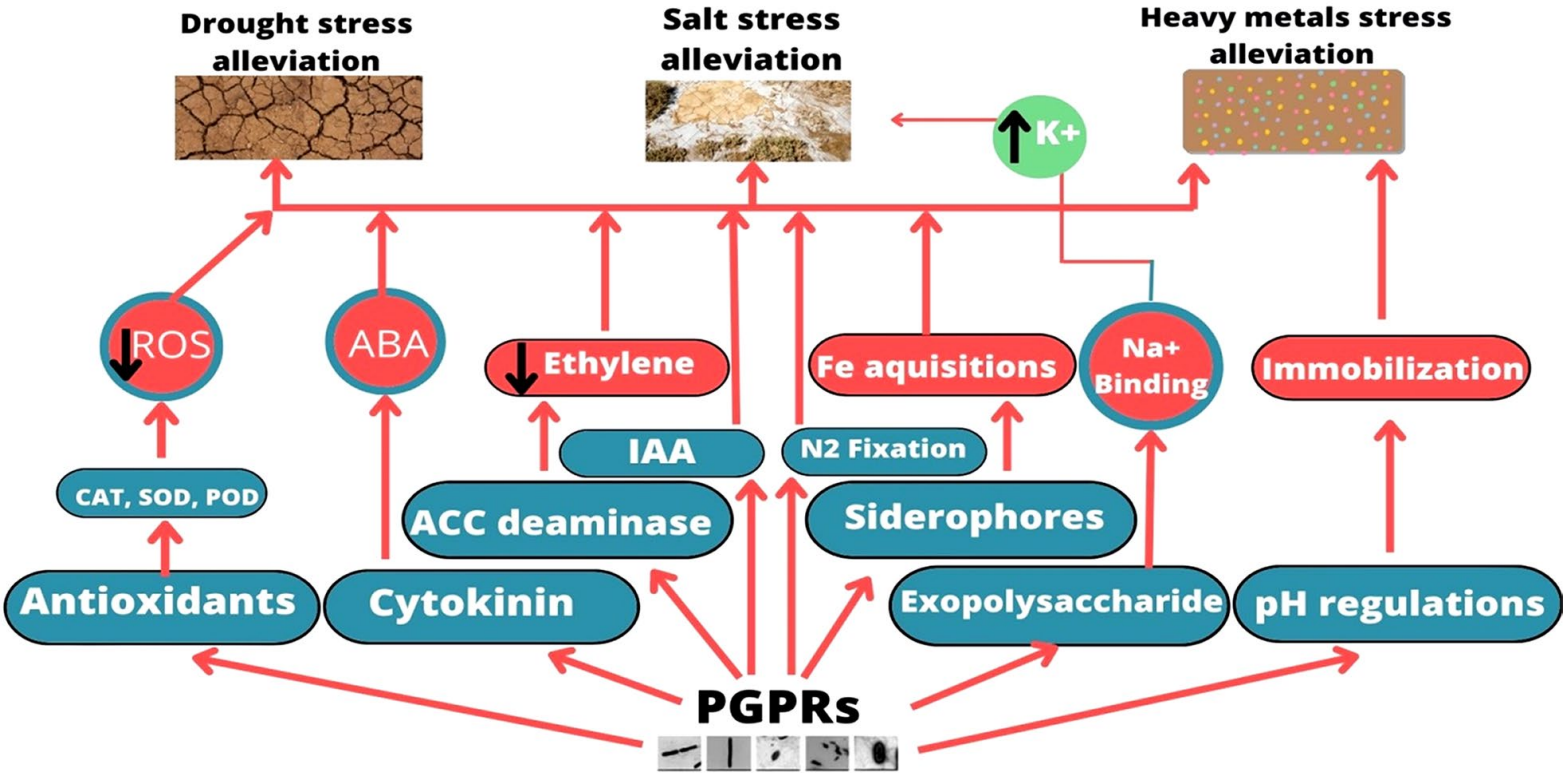
PGPR screening and its PGPR and molecular characterization

Various PGPR have been isolated and proven to alleviate various environmental stresses in plants and boost productivity. They may improve soil quality and boost plant productivity by direct and indirect mechanisms. Nitrogen fixation, phosphate and potassium solubilization, and production of growth-promoting phytohormones like indole acetic acid and siderophores are direct mechanisms through which PGPR perform these aforesaid functions; whereas, the indirect mechanisms involve production of lytic enzymes and antibiotics, lowering the soil pH, production of exopolysaccharides, etc. (Fig. 1). The effectiveness of PGPR for sustainable agro-ecosystem under normal and stress environments has been reviewed in many studies [15, 17, 18].

Biochar, a char produced by pyrolyzing organic materials particularly wastes under limited oxygen supply, has gained immense popularity for its vast range of uses like enhancing soil quality, soil carbon sequestration, adsorption and mitigation of organic and inorganic pollutants from aqueous and soil media, animal feedstock, etc. Multiple review articles and meta-analyses have summarized the positive effects of biochar on soil quality and agronomic productivity as well as the factors that contribute to the ameliorative role of biochar [19]. The biochars have also been used to alleviate various environmental stresses like salinity, drought, heavy metals, etc., from plants. This aspect has also been reviewed in multiple studies [20].

Sustainable food security is a major challenge in today’s world, more so in developing countries. The teeming millions in developing world, e.g., South Asia, South East Asia, and Africa, coupled with all around climate changes affecting agricultural operations and productivity are a major risk to sustainable food security [21]. According to Food and Agriculture Organization of the United Nations, the COVID-19 pandemic has worsened the food security such that over 2 billion people do not have enough food to eat [22]. Food and agriculture systems have already changed considerably, but more needs to be done in this changing global environment.

Different strategies are used to improve soil quality and increase the crop yields including land reforms, better water management, stress-tolerant varieties, increasing use of fertilizers, improved seeds, use of pesticides, genetically modified crops, plant growth regulators, and soil amendments; PGPR, biochar [4, 8, 23–25]. Given the trade-offs between food, fuel, housing and other uses of land, the quest for long-term, sustainable, eco-friendly and cost-effective techniques and tools for boosting soil quality and agricultural productivity has never been stronger and more urgent than today.

The agricultural productivity is reduced by different abiotic stresses such as salinity, drought, and heavy metal contaminants in soils among others [26]. The world’s land affected by salinity is 1125 million hectares, which is approximately 6% of the total global area including 20% of cultivated and 33% of the irrigated land. Soil salinization reduces productivity by up to 46 million hectares per year [27]. Soil salinity accounts for 1.5 million hectares of farmland from productions annually.

Crop and livestock production are water-intensive enterprises because agriculture is the largest consumer of water globally, accounting for 70% of global water returns [28]. Agricultural drought stress is one of the major abiotic stresses that are very common in semi-arid and arid areas around the world. Moreover, climate changes are exacerbating the droughts. Global demand for water for agriculture is expected to increase by 60% by 2025 [29]. Under drought stress, crop growth and yields are generally reduced due to low amounts of nutrients, poor photosynthesis and limited water supply [30]. Furthermore, drought accelerates the biological synthesis of ethylene in plants which inhibits root length and growth [31].

Another important abiotic stress is heavy metals in soils resulting in losses of agricultural productivity. Due to various natural and human activities, significant amounts of heavy metals are regularly added to the soil worldwide [32]. More than 10 million sites of soil contamination have been reported globally, with more than 50% of sites contaminated with heavy metals [33]. These heavy metals come into the soil from expanding industries, coal burning, wastewater irrigation, petrochemical spillage, coal combustion, animal manure, and sewage sludge [34]. Recent exponential increase in production and consumption of metal based nanoparticles has been found to enhance the soil contamination with heavy metals via sewage sludge applications. Moreover, increasing use of nano-metal-based fertilizers and pesticides is an emerging source of heavy metals in soils [35].

Recently, PGPR and biochar have been co-applied in various studies in order to improve soil quality and agronomic productivity under normal and stressed conditions. The explicit or underlying assumption in these studies has been that the biochar would increase nutrient availability and provide conducive habitat for the PGPR to flourish and in response the latter would perform their designated functions (phytohormone production, nutrient solubilization, etc.) at higher rates. These studies have been performed in stress-free as well as stressed soils. However, these studies have not been comprehensively synthesized and critically reviewed. This review paper aims to fill this gap. Moreover, we also present the future directions of research in order to optimally exploit the combined potential of PGPR and biochar for sustainable agro-ecosystem.

## Effect of co-application of biochar and PGPR on soil quality under normal conditions

Soil quality is a complex concept. The soils perform a variety of ecosystem services, which lead them to be defined from the point of view of those services [36]. From concurrent agricultural and environmental points of view, it is defined as the “the capacity of a soil to function within ecosystem and land-use boundaries to sustain biological productivity, maintain environmental quality, and promote plant and animal health” [37, 38]. The most commonly used chemical indicators of soil quality are soil organic matter, pH, and available macronutrients (nitrogen, phosphorus, and potassium). Similarly, the most commonly used physical indicators include water storage, bulk density, and structural stability, whereas the biological indicators include soil respiration, microbial biomass, nitrogen mineralization, and extracellular enzymatic activities [36]. The role of co-application of biochar and PGPR in improving the soil quality would be assessed based on these indicators in this review.

Various PGPRs co-applied with biochar are proposed as a good strategy to improve soil quality [39–41]. The presence of biochar can increase the efficiency of PGPR, as biochar provides a substrate to PGPR due to its high surface area and enriched nutrients for their survival [42]. In the following subsections, the effect of co-application of biochar and various PGPRs on soil quality and crop productivity has been reviewed.

### Effect on soil nutrients

A number of studies have assessed the effect of co-application of PGPR and biochar on soil quality defining physicochemical and biological properties of soils (Table 1). Co-application of biochar with PGPR has generally been found to increase the mineral nutrient content in soils when compared to sole application of either biochar or PGPR. For instance, combined use of biochar (2% w/w) and PGPR (*Paenibacillus polymyxa* and *Bacillus amyloliquefaciens*) showed 87% higher soil nitrate content than nitrogen only treatment [43]. Moreover, in the same study, soil urease activity in PGPR + biochar + nitrogen, was 34.20%, 13.51% and 44.78% higher than nitrogen only, biochar + nitrogen and PGPR + nitrogen, respectively. They found that soil NH_4_^+^-N contents in PGPR + biochar and biochar + nitrogen treatments was 136.83% and 82.07% higher than nitrogen only treatment. Jabborova et al. [44] evaluated the effect of co-inoculation of multifarious PGPRs (*Bradyrhizobium japonicum* and *Pseudomonas putida*) and different levels of maize biochar (1% and 3%) on soil nutrients. They found that co-application of the PGPR with 3% maize biochar increased available nitrogen, phosphorus, and potassium by 73%, 173%, and 17%, respectively, when compared to the 3% maize biochar only treatment. Ren et al. [45] found that using *Bacillus megaterium* (a nitrogen-fixing bacteria) with wheat-derived biochar increased nitrate, inorganic nitrogen, and total potassium in PGPR + biochar treatment by 68%, 45%, and 21%, respectively, than PGPR only and by 22%, 16%, and 30%, respectively, than biochar only treatment. Similarly, a PGPR *Bacillus megaterium,* when co-applied with biochar derived from agricultural waste, was found to increase organic carbon, available phosphorus, and available nitrogen by 16%, 79%, and 15%, respectively, in comparison to the control (no PGPR and no biochar) treatment. Saxena et al. [40] found that shoot nitrogen was 1.64 mg N g^−1^ shoot in soil treated with PGPR (*Bacillus* sp.) co-inoculated with biochar, which was significantly higher than that in sole applications of *Bacillus* sp. (1.24 1.64 mg N g^−1^ shoot) or biochar (1.31 mg N g^−1^ shoot). Overall these studies indicate that co-application of biochar and PGPR works in synergy to raise the nutrient level higher than the individual application of any of these. Biochars are rich in macro- as well as micro-nutrients. When applied to soils, they contribute nutrients to soils as a result of dissolution and decomposition under the influence of soil conditions and microbial activity [46]. The PGPR, particularly those solubilizing the organic phosphate, apparently accelerates the accrual of available phosphorus from biochar [47]. Consequently, freeing the soil microorganisms from investing on the acquisition some nutrients, the combined application of PGPR and biochar facilitates them to invest on acquisition of other nutrients thereby leading to enhancing enzymatic activity and release of other nutrients [48].

**Table 1:** Effect of co-applied PGPR and biochar under normal conditions on plant productivity and soil quality

Strain	PGPR traits	Biochar production and application rate	Crop	Experimental details	Impact on plant productivity and soil quality	References
*Micrococcus yunnanensis*	Organic and inorganic phosphate solubilizing, siderophore producing	Sugarcane bagasse derived biochar Pyrolyzed at 550 °C for 4 h Levels of BC application: 0, 1 and 2% w/w	Barley (*Hordeum vulgare*)	Green house experiment Plants were grown for 7 months	PGPR + 2% biochar treatment increased soil microbial biomass by 11% than un inoculated control (no PGPR, no biochar). Biological yield in PGPR + biochar was 42.1 g/pot, 38.9 g/pot in PGPR only and 36.3 g/pot in biochar only treatments 1000-kernel weight and phosphorus uptake was increased by 9% and 8% than PGPR alone treatment, respectively	[41]
*Paenibacillus polymyxa* and *Bacillus amyloliquefaciens*		Biochar was made from millet straw Level of BC application: 2% w/w	Tomato (*Lycopersicon esculentum*)	Green house Pot experiment Co-application of biochar and PGPR was evaluated in presence of absence of chemical N fertilizer	In combined PGPR + biochar + nitrogen application soil urease, ammonium, and nitrate was significantly increased between 14 and 45%, 137%, 31–87%, respectively, than nitrogen + PGPR and nitrogen + biochar Relative abundance of *Nitrospira* and *Bradyrhizobium* were increased in the soil The tomato yield was 32.45%, 45.69%, and 10.44% higher than those in the nitrogen, nitrogen + PGPR, and nitrogen + biochar treatments, respectively NUE (nitrogen use efficiency) increased by 11–18%	[43]
*Bacillus lentus*, and *Pseudomonas fluorescence*	Phosphate solubilizing	Biochar was prepared with cow manure, wheat straw and oak wood Pyrolyzed at 300–500 °C	Safflower (*Carthamus tinctorius*)	Farm assay was conducted for period of 2 years (2017 and 2018)	Highest grain yield of 1527 kg ha^−1^ was found in PGPR + biochar treatment Whereas grain yield by wheat straw biochar application alone was 1452 kg ha^−1^ and wood biochar was 1385 kg ha^−1^	[49]
*Bradyrhizobium japonicum* and *Pseudomonas putida*	NDA	Maize residues were pyrolyzed at 600 °C for 30 min	Soybean (*Glycine max*)	Green house experiment Control: soil with uninoculated, 1 and 3% biochar PGPR strains with 1 and 3% biochar Plant growth duration was 30 days	PGPRs + 3% biochar increased soil mineral nitrogen content by 73%, available phosphorus by 173%, and available potassium content by 17%, as compared to the 3% biochar only treatment It also increased protease activity by twofold, alkaline (1.25-fold) and acid phosphomonoesterase (1.52 fold) than PGPRs only and PGPRs with 1% maize biochar treatments Seed germination increased by 20%, root length by 76%, root dry weight by 56%, shoot length by 41% and shoot dry weight by 59% than the 3% biochar only treatment	[44]
*Bacillus megaterium*	Nitrogen-fixing, plant-probiotic	Wheat (*Triticum* L.) straw was carbonized at 600 °C for 3 h Application rate of biochar used: 20 t hm^−2^	Eucalyptus (*Eucalyptus globulus*)	Field experiment; PGPR(2 ml) + biochar (0.18 kg)	In PGPR + biochar treatment; nitrate, inorganic nitrogen, total potassium, EC (electrical conductivity) and soil water content were, respectively, 68%, 45%, 21%, 35% and 24% higher than PGPR only and, respectively, 22%, 16%, 30%, 5% and 18% higher than biochar only treatment	[45]
*Pseudomonas* sp.		Maize straw was pyrolyzed at 360 °C Application rate was 30 g/pot	Celery (*Apium graveolens* L.)	Solar greenhouse experiment Plants growth duration was 8 week of growth. High and low phosphorus (P) level and fungi was also combined with PGPR and biochar	PGPR + biochar increased P uptake by 24.5% and 72.1% than biochar only and PGPR only treatments Adding *Pseudomonas* sp. along with fungi and biochar increased root volume by 26–36% and 23–61% than fungi + biochar at high and low *P*, respectively It also increased root surface area by 27–73% at high and low *P* level, respectively, than using fungi + biochar	[39]
*Alcaligenes* sp.	Phosphorus and zinc solubilization, ACC deaminase activity, and IAA and siderophore production	Biochar source was maize straw Application level of biochar: 0.5 tons ha^−1^	Maize (*Zea mays* L.)	Field experiment; combined application of biochar, rock phosphate enriched compost humic acid and *Alcaligenes* sp. AZ9	PGPR + biochar increased bacterial population by 30%, and 15%; and microbial biomass carbon up to 12% and 6%, than PGPR and biochar only treatment Soil organic carbon and saturation percentage increased by 29% and 14%, respectively, than control (No additives) Adding PGPR with biochar increased shoot fresh biomass, shoot dry biomass, plant height, grain yield and 1000-grain weight by 9, 12, 6, 14 and 5%, respectively, than PGPR only Combined treatment increased 1000-grain weight by 10%, grain yield by 31%, and stover yield by 34% than control (No additives)	[50]
*Bacillus deuterium* and *Bacillus megaterium*	Nitrogen fixing, plant-probiotic	Wheat straw (*Triticum* L.) was pyrolyzed in a continuous carbonizer at 550 °C for 3 h Application level of biochar: BC0, BC20 and BC40 (0, 20, and 40 t hm^−2^, respectively)	Eucalyptus (*Eucalyptus globulus*)	Field experiment	Sucrase activity was 48%, 31% and 55% higher in PGPR + BC40 than the PGPR only, PGPR + BC20 and B20 treatments PGPR + BC40 significantly increased EC than PGPR, BC20 and PGPR + BC20 treatments	[51]
*Bacillus* sp. *Burkholderia* sp.	Phosphate solubilizing, siderophores and IAA production	Biochar was prepared from agricultural wastes Pyrolyzed at 600 °C		Pot experiment: plant growth duration was 90 days	*Burkholderia* + biochar treatment increased WHC (16%), EC (66%), SOC (16%), available phosphorus (79%), available nitrogen (15%) and hydrogenase activity (40%) than control treatment (without PGPR and biochar) Combined application of biochar and PGPR increased germination (18.06%), shoot fresh and dry biomass (72.5% and 45.5%, respectively) and root length (113%) over control treatment (without PGPR and biochar)	[52]
*Lysinibacillus fusiformis* and *Bacillus subtilis*	Phosphate solubilizing	Biochar was prepared from woody sawdust Pyrolyzed at 350 °C for 10 min Application level of biochar (Bagasse and sawdust biochar): 1% (30/3 kg soil)	Maize (*Zea mays* L.)	Green house pot experiment Plant growth duration was 65 days	36.2% more nitrogen and 58.3% more phosphorus was found in PGPR + biochar treatment than untreated control PGPR + sawdust biochar increased root and shoot length by 45% and 64% than the uninoculated and untreated control PGPR + biochar increased plant phosphorus (72.5%), nitrogen(32.8%), and potassium (42.1%) than untreated control	[53]
*Stenotrophomonas* sp.	Nitrogen-fixing bacteria	Biochar was prepared from empty fruit bunch of oil palm Pyrolyzed at 350–450 °C Application level of biochar: 0, 0.25, 0.5, 0.75 and 1% of soil (lab experiment) Biochar levels: 0, 5, 10, 15 and 20 t/ha (Green house experiment)	Sweet corn	Lab as well as greenhouse experiments	PGPR + 0.5%biochar treatment showed pH 5.7 (highest) and in PGPR treatment it was 4.7. Nitrogen, phosphorus and potassium were 94.5%, 65.93% and 75.30% higher in PGPR + biochar, respectively, than PGPR treatment Plant weight increased from 48.5 g plant^−1^ (in 5 t ha^−1^ biochar) to 61.4 g plant^−1^ (in PGPR + 5 t ha^−1^ biochar)	[54]
*Pseudomonas* species, *Azotobacter chroococcum* and *Azospirillum brasilense*	IAA producing and phosphate solubilizing	Biochar was produced from rice husk Application level of biochar: 3.6 g kg^−1^ soil	Rice (*Oryza sativa*)	Pot experiment; rice grown in alluvial soil in Kharif season	Rice husk biochar + PGPR increased growth and dry matter yield of rice than untreated control	[55]
	Nitrogen fixing and phosphate solubilizing	Biochar was produced from soft wood chips Pyrolyzed at 500 °C Application level of biochar: 20 Mg ha^−1^	Switchgrass (*Panicum virgatum* cv. Cave-in-Rock)	Field experiment; Plant growth duration was 3 years at two research sites	Plant height increased from 1 to 8% in both biochar + PGPR (nitrogen-fixing) and biochar + PGPR (phosphorus solubilizing) bacteria treatment than control (No PGPR, no biochar)	[56]
*Bacillus* sp.	Phosphate solubilizing	Biochar: application rate was: 15 g kg^−1^ of soil	French beans (*Phaseolus vulgaris*)	Pot experiment; plant growth duration was 8 weeks	PGPR + biochar treatment, increased no. of phosphate solubilizing bacterial count 2.95 ± 0.11 × 10^6^ in the rhizosphere of plants than the PGPR 1.82 ± 0.06 × 10^6^ The shoot biomass (3.22 g), root length (14.88 cm) and root biomass (1.85 g) were significantly higher in the PGPR + biochar treatment compared to PGPR treatment where they were 2.34 g, 13.13 cm, and 1.31 g, respectively, as well as than biochar treatment where they were 3.19 g, 13.12 and 1.75, respectively PGPR + biochar increased percent nitrogen content 1.64 shoot/g then PGPR treatment 1.24shoot/g and biochar treatment 1.31 shoot/g	[40]

### Effect on water holding capacity of soil

The biochar has potential to improve water holding capacity of soils, particularly for coarse-textured ones, thanks to its large surface area-to-volume ratio. A number of reviews have compiled studies on this question [57, 58]. Some of the studies testing co-application of biochar and PGPR have also reported the ameliorative effect of biochar on water holding capacity. Co-application of a nitrogen-fixing PGPR, *Bacillus megaterium*, along with wheat-derived biochar increased soil WHC by 24% and 18% than PGPR only and biochar only treatments, respectively [45]. Although the PGPR alone has never been reported to ameliorate water holding capacity nor water content of a soil, they may enhance drought tolerance of crop plants [31]. However, it must be expected that the enhanced WHC, thanks to biochar, would synergize with PGPR given that the nutrient cycling, soil organic matter decomposition, and microbial signaling becomes better under optimum moisture conditions [59, 60]. It must be noted that this indirect benefit of co-applying biochar with PGPR has not been explored so far.

**Table 2:** Effect of co-applied PGPR and biochar on plant productivity and soil quality under environmental stressors

Strain	PGPR trait	Biochar production and application rate	Crop	Experimental details	Effect on plant productivity and soil fertility	References
*Pseudomonas putida*, *Planomicrobium chinense*		Produced from plant leaves Pyrolyzed at 300–400 °C for 12 h	Soybean (*Glycine max*)	Pot experiment; Drought applied after 5 weeks of germination, with holding water supply for 4 days	In PGPR + biochar, leaf water content by 9%, root to shoot ratio by 54% and proline content by 65.5% increased over control Similarly, nitrogen, phosphorus, potassium were increased by 9.5%, 24% and 26%, respectively, than control	[61]
*Pseudomonas* sp. and *Staphylococcus* sp.		Produced from *Morus alba* L. wood in oxygen-limited conditions	*Brassica napus* L.	Field experiment; drought stress applied for 15–30 days	Co-application improved plant antioxidant enzyme activity including ascorbate peroxidase (APX) and catalase (CAT) Also enhanced the content of photosynthetic pigments such as chlorophyll pigments, anthocyanin content and carotenoids content	[62]
*Sphingobacterium pakistanensis*, *Cellulomonas pakistanensis*		Produced from Wood residues of mulberry plant (*Morus alba* L.) combustion at 500–750 °C Application rate was 5%	*Vicia faba*	Pot experiment; drought induction was 13 days and 26 days	In PGPR + biochar, relative water content improved by 35.82–54.34% Similarly, photosynthetic pigments and proline improved by 58.33–173.8% and 46.58–86.62%, respectively	[63]
*Azotobacter chroococcum* and *Pseudomonas koreensis*	IAA producing and phosphate solubilizing	Biochar was produced from rice husk and corn stalk at the ratio (1:1) Pyrolyzed at 350 °C for 3 h Application level of biochar was: 10 ton/ha	Maize (*Zea mays* L.)	Field experiment; irrigation with saline water Plant growth duration was till its maturity	PGPR + biochar treatment increased soil urease and dehydrogenase activity by 26.6% and 33.5%, respectively, than PGPR and biochar treatment. EC decreased by 9% than PGPR treatment and by 5.6% than biochar treatment Similarly, no. of grains ear^−1^, 100-grain weight, grain yield and stover yield increased by 1.9%, 17%, 24.60% and 5.23%, respectively, than PGPR treatment, and by 1.1%,13.44%, 9.5% and 4.38%, respectively, than biochar treatment	[64]
*Cellulomonas pakistanensis* and *Sphingobacterium pakistanensis*	–	Biochar was produced from wood of *Morus alba* Pyrolyzed at between 900 and 1100 °C Applications of biochar level were 0 and 5% w/w	Pea and bean family (*Vicia faba*)	Pot experiment; Drought applied for 13 and 26 days Plants were grown for 26 days	PGPR + biochar treatment positively ameliorated fresh and dry weight of leaves by 28.57 and 10.47%, roots 36.36 and 14.28% and shoots by 16 and 10% than sole biochar and PGPR treatments, respectively	[65]
*Alcaligenes faecalis*, and *Bacillus amyloliquefaciens*	ACC deaminase producing	Biochar was made from vegetables and fruits Pyrolyzed at 450 °C for 2 h Application of biochar level was: 0.5%	Mint (*Mentha piperita* L.)	Pot experiment: lead stress: 250 mg Pb kg^−1^soil	*Alcaligenes faecalis* strain + compost-mixed biochar treatment showed significant results in improving plant chlorophyll content (37%), root dry weight (58%) and nitrogen (46%), phosphorus (39%), and potassium (63%) in leaves of mint than untreated control Lead (artificially induced) uptake also decreased in spinach roots by 43% and potassium uptake increased by 10.5% over untreated control	[66]
*Pseudomonas* *aeruginosa*, *Enterobacter cloacae*, *Achromobacter xylosoxidans* and *Leclercia adecarboxylata*	Drought tolerant and ACC-deaminase producing	Biochar was produced from timber waste Pyrolyzed at 389 °C for 80 min. Application of biochar levels were: 0.75 and 1.5% w/w	Maize (*Zea mays* L.)	Drought/moisture conditions: 70% of field capacity (optimum moisture), 50% FC (mild) and 30% FC (severe drought) Duration of plant growth was 3 months (Harvesting at maturity)	*A. xylosoxidans* + 1.5% biochar treatment showed 43% and 25 increase in grain yield pot^−1^ than *A. xylosoxidans* and 1.5% biochar using alone *A. xylosoxidans* + 1.5% biochar showed 19 and 6% higher photosynthetic rate, 30 and 7% higher transpiration rate, and 16% and 7% higher stomatal conductance, respectively, than *A. xylosoxidans* and 1.5% biochar using alone under severe drought *P. aeruginosa* + biochar decreased electrolyte leakage by 28% and 4% than sole application of *P. aeruginosa* and biochar, respectively	[67]
*Burkholderia phytofirmans*	Siderophore-producing endophytic	Biochar was produced from tree twigs feedstock Pyrolyzed at 400 °C for 40 min Application of biochar level was 1%	Quinoa (*Chenopodium quinoa*)	Salinity level: EC 20 dS/m	PGPR + biochar treatment showed 2.73 × 10^5^ CFU/g in rhizosphere, 9.92 × 10^4^ CFU/g interior root and 1.9 × 10^4^ CFU/g interior shoots bacterial population than the 5.73 × 10^5^ CFU/g in rhizosphere, 4.53 × 10^5^ CFU/g interior root and 9.92 × 10^4^ CFU/g interior shoots bacterial population found in biochar treatment. Plant height, root dry weight, shoot dry weight, grain yield, photosynthetic rate, stomatal conductance increased by 17, 26, 10, 5, 5, 16 and 12%, respectively, than PGPR only treatment	[68]
*Alcaligenes faecalis*, and *Bacillus amyloliquefaciens*	ACC deaminase producing	Biochar was made from vegetables and fruits Pyrolyzed at 450 °C for 120minuts Application of biochar level was:0.5%	Spinach (*Spinacia oleracea*)	Pot experiment: lead stress: 250 mg Pb kg^−1^ soil Compost (mixed fruits) mix biochar (vegetable waste) with 1:1	Treatment ACC deaminase producing PGPRs and compost-mixed biochar showed 13.5% less uptake of Pb in leaves of mint plant than the soil without PGPR and biochar under lead stress	[69]
*Bacillus* sp.	Cd-immobilizing, IAA producing and phosphate solubilizing	Biochar was produced from coconut shell. Pyrolyzed at 800 °C for 6 h Application of biochar level was: 5 g/100 ml suspension	Ryegrass (*Lolium perenne*)	Pot experiment; Cd polluted soil was used. Plant growth duration was till at maturity	PGPR + biochar treatment showed *Bacillus* sp. increased by 7.46% than PGPR only treatment PGPR + biochar treatment showed dehydrogenase 4.61 times more than biochar treatment 2.47times HOAc-extractable Cd decreased in soil by 11.34% in than biochar 4.49% and PGPR 6.05% treatments, respectively. PGPR + biochar treatment = ryegrass biomass 1.96 g found than biochar treatment only 0.42 g Lowest Cd concentration in ryegrass 5.45 mg kg^−1^ found in PGPR + biochar than biochar, PGPR and soil without PGPR and biochar treatments	[70]
*Bacillus* sp*.*	Ni, Pb and Cr tolerant IAA and ACC deaminase producing	Application level was: 1%	Wheat (*Triticum aestivum* L.)	Green house pot experiment K_2_Cr_2_O_7_ solution was applied at the rate of 2.5 mg/Kg with irrigation	PGPR + biochar treatment increased shoot and root length by 22–23.4%, and maximum increase in chlorophyll and SOD was 28–40%. Combined treatment also maintained proline and sugar contents by 20.5% and 9.6% In dry biomass, Cr concentration was 0.28 ± 1.01 mg/kg than uninoculated control 0.05 ± 1.01 mg/kg	[71]
*Enterobacter* sp*.*	Cd tolerant	Biochar was produced from paper and pulp waste Pyrolyzed at 450 °C Application of biochar levels were: 0 and 10 g kg^−1^	Rapeseed (*Brassica napus*)	Pot experiment;. Soil was spiked with Cd at the at the 2 levels 0 and 80 mg kg^−1^ dry soil by using Cd (NO_3_)_2_ Plant growth duration was 60 days	PGPR + biochar treatment under Cd stress decreased Cd concentration by 45.6% in soil than PGPR and biochar. Bacterial population was 4.5 × 10^5^ in co-applied, whereas it was 1.8 × 10^5^ in biochar only treatment Under Cd stress, PGPR + biochar treatment decreased Cd by 40.1 and 38.2% in root and shoot than PGPR treatment, by 16.8 and 16.9% than biochar treatment, and by 23.4 and 21.3%, respectively, than control (No PGPR, no biochar)	[16]
*Agrobacterium fabrum* and *Bacillus amyloliquefaciens*	ACC deaminase producing	Biochar was produced from timber waste Pyrolyzed at 389 °C for 1 h and 20 min. Application of biochar levels were: 0 and 1.5%	Wheat (*Triticum aestivum* L.)	Field experiment; drought stress induced by skiping (4I control, 3I mild and 2I severe drought). Plant growth duration was 120 days.	*Bacillus amyloliquefaciens* + biochar treatment showed 34% and 24% increase in plant height, 25% and 8% in root length, and 5% and 2% in spike length, respectively, than *Bacillus amyloliquefaciens* and biochar using alone under severe drought. *Agrobacterium fabrum* + biochar showed increase in 1000-grain weight by 13% and 2% than *Agrobacterium fabrum* using alone and with biochar under severe drought	[72]
*Pseudomonas koreensis* and *Bacillus coagulans*	IAA producing and inorganic phosphate solubilizing	Biochar was produced from rice husk and corn stalk at the ratio (1:1) Pyrolyzed at 350 °C Application of biochar rate was 2 kg m^−2^	Rice (*Oryza sativa* L.)	Field Experiment; soil was salt affected, water deficit conditions were created by irrigation every 6, 8 and 10 days Duration of plant growth was till its maturity	At irrigation after 6 days improved soil moisture content and minimum level of SAR, Na^+^ and EC were found using combined treatment PGPR + biochar than using PGPR and biochar alone Grain yield increased by 9.9% and 5.5% and straw yield by 6.68% and 3.78% in PGPR + biochar treatment than PGPR and biochar only treatment. Relative water content and stomatal conductance of rice leaves were enhanced by PGPR + biochar by 8.2% and 12.19% than PGPR and biochar only treatment, respectively. Similarly, 1000-grain weight was increased by 6–22% and proline content in leaves decreased by 38.86% and 22% than PGPR treatment and biochar treatment, respectively	[60]
*Leclercia adecarboxylata*, *Agrobacterium fabrum*, *Bacillus amyloliquefaciens*, *Pseudomonas aeruginosa*	ACC deaminase producing	Biochar was produced from timber waste Pyrolyzed at 389 °C for 1 h and 20 min. Application of biochar levels were 0, 1 and 1.5%	Wheat (*Triticum aestivum* L.)	Pot experiment; drought stress applied by maintaining 50% and 30% of field capacity. Plant growth duration was 50 days	*B*. *amyloliquefaciens* + 1.5% biochar treatment under stressed conditions increased the chlorophyll a by 114%,, chlorophyll b by 123%, photosynthetic rate by 118%, transpiration rate by 73%, 100-grain weight by 59%, and grain N, P and K up to 58%, 18% and 23%, respectively, compared to control	[73]
*Micrococcus* sp. and *Arthrobacter* sp*.*	IAA producing and cadmium-resistant bacteria	Biochar was produced from cassava stem (*Manihot esculenta* L. Crantz). Pyrolyzed at 300 °C for 120 min Application of biochar level was: 0.2%	*Chlorophytum laxum*	Green house pot experiment; Cd containing soil (pristine) Plant growth duration was 9 weeks	*Micrococcus *sp. + biochar treatment increased root dry weight by 1.2 and 1.1 fold at 6 and 9 weeks of harvesting, respectively, than the PGPR and biochar treatments under Cd stress	[74]
*Pseudomonas fluorescens*		Biochar was produced from azolla biomass Pyrolyzed at 600 °C under oxygen-limited conditions Application of biochar level was:0 and 1%	*Rosemary *(*Rosmarinus Officinalis* L.)	Green house pot experiment Calcareous soil was used Plant growth duration was 6 month	PGPR + biochar treatment increased microbial biomass carbon by 34.9% than control Nitrogen, phosphorus and potassium content in PGPR + biochar treatment increased by 22%, 10% and 9.5% than PGPR treatment and by 8.6%, 13% and 13.5%, respectively, than biochar treatment Similarly, PGPR + biochar treatment increased hoot fresh weight increased by 34.7%, fresh weight of roots by 27%, plant height by 18.2% than untreated control	[75]
*Serratia odorifera*	ACC deainase producing and drought tolerant	Biochar produced from algal biomass. Pyrolyzed at 300 °C for 60 min Application of biochar level was 4% w/w	Maize (*Zea mays* L.)	Pot experiment; drought stress induced by maintaining field capacity (FC) at 75% and 50% Plant growth duration was two months	PGPR + biochar increased pH by 7 and 5%, EC by 34 and 13%, nitrate by 57 and 34%, phosphorus by 54 and 49%, extractable potassium by 30 and 15% and organic matter by 69 and 21% under 50% field capacity than PGPR and biochar treatments using alone. Similarly, plant height increased by 38 and 16%, shoot fresh weight by 29 and 17%, shoot dry weight by 44 and 24%, root fresh weight by 60 and 27%, root dry weight by 84% and 24%, and root length by 47% and 32%, respectively, than PGPR and biochar treatments using alone under 50% field capacity	[76]
*Azospirillum*	Free-living, mutualistic nitrogen fixators and phosphate solubilizers	Biochar was produced from grain husks and paper fiber sludge. Pyrolyzed at 450–500 °C for 20 min Application of biochar level was: 3 t ha^−1^	Maize (*Zea mays* L.)	Field trial; area was prone to drought Plant growth duration was 5 months	PGPR + biochar treatment increased substrate induced respiration in acidic soil by up to 100% and 50% in calcareous soil than unamended control (No PGPR, no biochar) PGPR + biochar treatment increased above ground biomass by 91% than unamended control	[77]
*Pseudomonas fluorescens*	ACC deaminase, exopolysaccharides and osmolyte producing	Biochar was produced from pine wood Pyrolyzed at 300 ºC Application of biochar levels were: 0 and 2% w/w	Cucumber (*Cucumis sativus*)	Pot experiment; water deficit conditions maintained by maintaining field capacity (FC) at 50, 75, 100% Duration of plant growth was 35 days. Compost used was 5% level	*Pseudomonas fluorescens* + biochar at 50% field capacity showed 70% more root colonization in soil than PGPR only treatment PGPR + biochar treatment at 50% field capacity showed increases in shoot length, shoot fresh weight, root length, and root fresh weight by 10%, 10%, 29% and 16%, respectively, than the 2% biochar only treatment. Also chlorophyll content and relative water content increased by 5% and 6% than 2% biochar only treatment	[59]
*Bradyrhizobium japonicum*	Nitrogen fixing	Biochar was produced from woody biomass Produced in closed reactor. Application of biochar levels were: 1, 2.5, and 5%	Mung bean (*Vigna mungo*)	Green house pot experiment; plant growth duration was 12 weeks	Biochar at 2.5% with PGPR was the most effective treatment that lead to 40% increase in soil microbial biomass carbon, 2.5% biochar with PGPR increased plant height and root length than other treatments	[78]
*Pseudomonas* sp.	Phosphate solubilizing	Biochar was produced from poplar saw dust Application of biochar level was: 5 g/Kg of soil	Maize (*Zea* *mays* L.)	Pot experiment; in growth chamber with anoxic conditions, induced salinity stress using NaCl at 150 mM rate Biochar + fertilizer used in ratio (5:1)	PGPR + biochar treatment showed soil moisture content increased almost 20% than in PGPR treatment. Moreover, *Pseudomonas* sp. + biochar treatment 30% increased calcium and potassium content in aerial part of maize than PGPR treatment under saline conditions PGPR + biochar treatment increases peroxidase activity by 50% and decreased proline content (leaves) by 20% than PGPR treatment	[79]
*Burkholderia phytofirmans* and *Enterobacter* sp.	Endophytic ACC deaminase and exopolysaccharide producing	Biochar was produced from hard wood (80%) and soft wood (20%) mixture Pyrolyzed at 500 °C. Application of biochar levels were: 0 and 5%	Maize (*Zea mays* L.)	Green house pot experiment Induced salinity: with irrigation water containing 0 and 25 mM NaCl solution, respectively Duration of plant growth was 2 months	Strain *Enterobacter* + 5% biochar treatment showed an higher colonizing efficiency (6.0 CUF g^–1^ DW) in rhizosphere than another strain *Burkholderia phytofirmans* + biochar in saline soil *Enterobacter* + 5% biochar treatment and *B. phytofirmans* + 5% biochar treatment reduced Na^+^ uptake by 25% and 8%, respectively, than biochar only treatment	[80]

### Effect on indigenous soil microbial communities

Many physicochemical properties of soil are improved by biochar, which ultimately facilitate the working of indigenous soil microbial communities. For instance, biochar may improve water holding capacity, pH (liming effect), and substrate and nutrient availability, which may lead to increase in microbial biomass, abundance and diversity [81, 82]. However, co-application of PGPR along with a biochar may also ease the nodulation process and improve symbiotic performance of a rhizobium [83]. Moreover, biochar has also been shown to improve the nodulation of the natural rhizobia with plants. This is due to the improvement in aeration by biochar that provides more air to nodule bacteria, which may survive for long on the porous surface of a biochar before ultimately colonizing a root [84, 85]. Similarly, adding biochar may further improve the mutualistic relationship of extant microbes for the benefit of plants. For instance, adding biochar and *Pseudomonas* sp. increased root colonization by arbuscular mycorrhizal fungi when compared to sole addition of *Pseudomonas* sp. and/or AMF. The phosphate-solubilizing *Pseudomonas* sp. enhanced available phosphorus in the soil presumably by solubilizing it from the biochar thereby leading to enhanced root colonization and overall growth of the plant [39]. The combined application of biochar and PGPR may enhance the general abundance of certain microbial groups in soil, which contribute to overall improved soil quality. For example, an *Alcaligenes* sp. strain in interaction with a maize-stalk-derived biochar increased the population of soil bacteria by 30% when compared to sole application of *Alcaligenes* sp. and by 15% when compared to biochar only treatment. Similarly, inoculation by *Bacillus megaterium* of a eucalyptus plantation along with addition of wheat-derived biochar significantly improved the microbial community in the soil, thereby leading to improved nutrient availability. The authors attributed this increase in beneficial microbes to the enhanced soil organic matter content and its decomposition due to interactive effect of biochar and the inoculant [45].

### Effect on intra- and extra-cellular enzymes

The potential beneficial effects of combined application of biochar and PGPR have also been assessed and reported by studying various intra- and extracellular enzymes. Combined application of a biochar with a nitrogen-fixing *Bacillus deuterium* increased soil sucrose activity to 4.8 mg.g^−1^ and 3.31 mg.g^−1^ from 2.48 mg.g^−1^ in PGPR only treatment [51]. Soil urease activity was 44.78%, and 13.51% higher while using *Paenibacillus polymyxa* and *Bacillus amyloliquefaciens* with biochar (2% w/w) treatment than that in the sole PGPR and biochar treatments, respectively [43]. Jabborova et al. [44] found the increase in protease (twofold), alkaline (1.3 fold) and acid phosphomonoesterase (1.5-fold) using co-inoculation of PGPRs (*Bradyrhizobium japonicum* and *Pseudomonas putida*) with 3% maize biochar than PGPRs only and PGPRs with 1% maize biochar. Similarly, synergistic use of *Bacillus subtilis* with cotton-derived biochar was found to significantly enhance the invertase and catalase activities in soil than the biochar only treatment [86]. Co-application of *B. japonicum* and *P. putida* with the biochar (10 t ha^−1^) has been reported to increase the activity of different enzymes like FDA activity, alkaline phosphomonesterases and proteases in the soil than biochar only and the uninoculated control (no PGPR, no biochar) [87]. Overall PGPR in combination with biochar have found to increase soil sucrase, urease, protease, invertase, catalase, alkaline and acid phosphomonoesterase enzymatic activity. These enzymes stimulate biochemical processes in soil ecosystem and can define direction and intensity of nutrient transformation processes in soil, thus ensuring enhanced soil fertility. Enhanced activity of the enzymes in soil by PGPR and biochar has linear relationships with soil nutrients [43, 50]. It must be noted that the effect of co-application of biochar and biochar on important N-cycling enzymes, leucine aminopeptidase and N-acetyl-glucosaminidase, has not yet been explored. These enzymes catalyze complex proteinaceous materials in soil [88]. Given that the biochar are complex organic materials packing organic proteins, the activity of these enzymes in the presence of PGPR could reveal the extent of accrual of mineral nitrogen from the added biochars.

## Effect of co-application of biochar and PGPR on agricultural productivity under normal conditions

Sustainable agriculture requires that crops grow with a low rate of agrochemical application possessing better nutritional values and disease resistance. Widespread use of expensive agrochemicals in agriculture has led to the use of more sustainable alternatives, such as PGPR and biochar in recent decades [89, 90]. Both PGPR and biochar have been extensively documented for their positive effects on plants. But in recent years the combined use of PGPR and biochar has also proved to be more effective in plant production than using PGPR or biochar separately. Various studies have reported positive effects of combined application of PGPR and biochar [29, 70, 71, 91]. For instance, a PGPR *Micrococcus yunnanensis*, when co-applied with 2% biochar, increased the yield to 42.1 g pot^−1^ from 38.9 g pot^−1^ when applied alone or from 36.3 g pot^−1^ when biochar was applied alone [41]. Co-application of both also induced a 9% increase in 1000-kernel weight than *Micrococcus yunnanensis* only and 8% increase in phosphorus uptake than the 2% biochar alone treatments. Yuan et al. [43] reported an increase in tomato yield in co-applied PGPR (*Paenibacillus polymyxa and Bacillus amyloliquefaciens*) strains with 2% biochar derived from millet straw and nitrogen fertilizer. They recorded 32.45%, 10.44% and 45.69% higher yield in PGPR + biochar + nitrogen than nitrogen only, biochar + nitrogen and PGPR + nitrogen treatments, respectively. Jabborova et al. [44] found seed germination increased by 20%, root length by 76%, root dry weight by 56%, shoot length by 41% and shoot dry weight by 59% with co-inoculation of *Bradyrhizobium japonicum* and *Pseudomonas putida* with 3% maize biochar than in 3% biochar only treatment. Similarly, combined application of *Alcaligenes* sp. with 0.5 t ha^−1^ maize biochar enhanced the shoot fresh biomass, shoot dry biomass, plant height, grain yield, and 1000-grain weight by 9, 12, 6, 14 and 5%, respectively, than PGPR alone [50]. A 3% increase in plant height, 11% in shoot weight and 61% in number of nodules of cowpea plant were found by using biofertilizer (made from consortium of *Bacillus thuringiensis, Pseudomonas putida* and *Klebsiella variicola* PGPR strains) in combination with biochar than the biofertilizer only [92]. Combination of PGPR(s) with biochar has also been tested under reduced fertilizer regime in an effort to minimize the greenhouse gas emissions associated with fabrication of ammoniac fertilizers and their volatilization. For instance, combined application of PGPRs, i.e., *Enterobacter*, *Pseudomonas*, *Azospirillum*, *Agrobacterium* and biochar raised the wheat yield to 5.04 t ha^−1^ than 2.56 t ha^−1^ PGPR only and 3.16 t ha^−1^ in biochar only treatments [93]. Similarly, combining *Bacillus* sp with biochar in French beans increased shoot biomass from 2.34 g pot^−1^ to 3.22 g pot^−1^, root length from 13.33 cm to 14.88 cm, and root biomass from 1.31 g pot^−1^ to 1.85 g pot^−1^, respectively [40]. Overall, these studies show that the combined application of PGPR and biochar can increase seed germination, plant growth such as plant height, shoot length, shoot dry weight, shoot biomass, root length, root dry weight, root biomass and plant yield than the individual application of PGPR or biochar. This combination may work in two ways. In the direct mechanism, the usual production of phytohormones by the PGPR like indole acetic acid, siderophores, etc., and increase in soil nutrients via phosphate solubilization and N_2_ fixation leads to higher plant growth and yield. Indirectly, the presence of biochar may facilitate the survival of the PGPR in higher numbers in addition to providing them nutrient rich substrate thereby leading enhanced performance by the PGPR ultimately resulting in higher plant production [94].

## Co-application of biochar and PGPR under environmental stressors

**Fig. 2:**
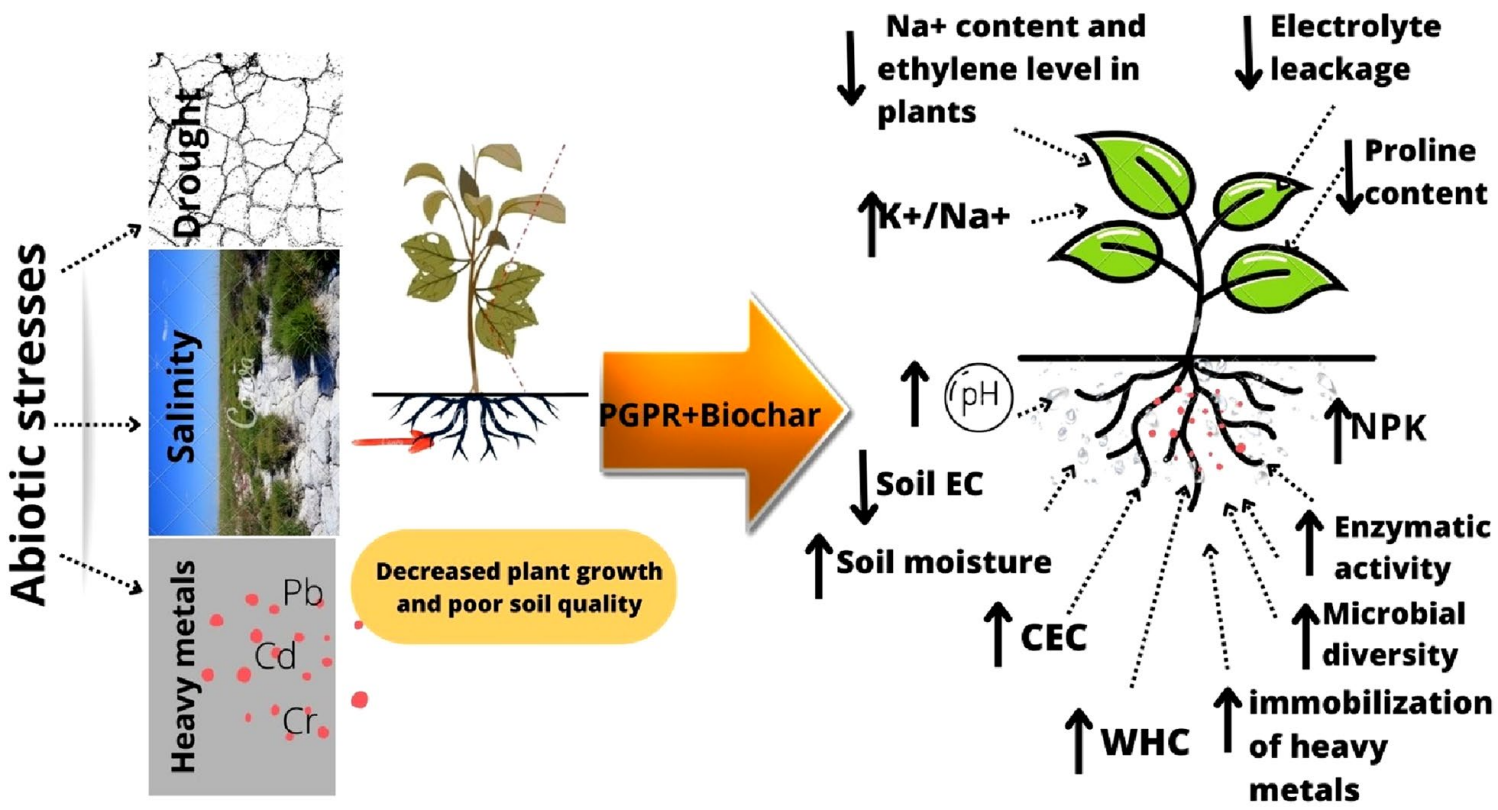
Effect of PGPR and biochar on plant growth and soil quality under different environmental stresses

The PGPR are known since long to help alleviate multitude of environmental stressors that hamper plant growth and development. They have been proven very effective against drought, salinity, heavy metal contamination (Fig. 2).

For instance, the potential of PGPR to secrete exopolysaccharides under dry conditions help induce drought tolerance in plants [95]. Under saline conditions, they could enhance potassium uptake at the cost of sodium thereby mitigating direct adverse effects of soil salinity, increase water uptake, reduce stomatal conductance, and antioxidant enzyme activities. All of these changes help plants to grow better under saline conditions [96]. Similarly, the PGPR have been found to immobilize and reduce uptake of heavy metals by plants in addition to improving the overall nutrient uptake thereby alleviating the heavy metal induced toxicity [97]. These findings have been reviewed in a number of papers [95, 98].

Biochar has also been shown to enhance salinity tolerance, alleviate drought stress, and mitigate the toxicity induced to plants by inorganic and organic soil pollutants. Drought stress alleviation in biochar-amended soils occurs through enhanced water holding capacity thanks to large surface area-to-volume ratio of biochar [80]. Similarly, decrease in osmotic stress thanks to improved soil water content in addition to reduced Na^+^ uptake due to Na^+^’s transient binding on sorption sites on biochar alleviate soil salinity stress for plants in biochar-amended soils [80]. Sorption is also the major mechanism through which biochar alleviates toxicity stress of organic and inorganic heavy metals. All these uses of biochar against different environmental stressors have been reviewed in multiple articles [49, 99, 100]. Recently, some studies have explored the potential of co-application of PGPR and biochar to alleviate the environmental stressors for plant growth with the assumption that both the additives would act synergistically (Table 2). Although the mechanistic synergism between the two, i.e., PGPR and biochar, has not been actively explored in these studies, synergies have indeed been found. The following sections would narrate these studies.

### Effect of co-application of PGPR and biochar on soil quality under environmental stressors

The combined use of PGPR and biochar perform multiple functions in alleviation of drought stress thereby leading to improved soil quality (Table 2). Both seem to work in tandem to improve the soil functions thereby alleviating the drought stress. For instance, combined application of algal biochar (4% w/w) and a PGPR *Serratia odorifera* to maize, when moisture content was 50% of the field capacity, significantly improved pH by 7 and 5%, EC by 34 and 13%, nitrate by 57 and 34%, phosphorus by 54 and 49%, extractable K by 30 and 15%, and organic matter by 69 and 21% in comparison to biochar alone and PGPR alone treatments, respectively [76]. Similarly, Nafees et al. [65] co-applied *Cellulomonas pakistanensis* or *Sphingobacterium pakistanensis* with biochar to *Vicia faba* growing on induced drought stress. They found that the combined application increased the water-use efficiency by 43.62%. In another study, soil moisture content was significantly higher in combined application of *Pseudomonas* sp. and biochar derived from poplar saw dust than sole application of PGPR or biochar [79]. The emerging pattern from these studies suggest that the enhanced water holding capacity and concurrent reduction in drought stress bolsters the survival and abundance of the PGPR, which in turn, perform their functions better [76].

As far as the soil quality is concerned, salinity reduces microbial activity and biomass in addition to changing the microbial community structure in soil [101]. Moreover, in saline conditions K^+^ transport channels are overtaken by Na^+^ leading to lower and reduced plant growth [102]. However, co-application of PGPR and biochar under saline conditions has been shown to induce salt tolerance and plant growth mainly by reducing Na^+^ uptake and improving K^+^/Na^+^ ratio. For instance, co-application of either of the two endophytic PGPRs, *Burkholderia phytofirmans* or *Enterobacter* sp*,* with biochar significantly mitigated the salinity stress in maize by reducing the xylem Na + uptake [80]. Similarly, co-application of *Pseudomonas koreensis* and *Bacillus coagulans* PGPRs with biochar significantly increased the K^+^ and K^+^/Na^+^ ratio thereby leading to lowered salinity stress in rice plants [60]. In the same study, the sodium adsorption ratio and Na^+^ in soil solution were also decreased by the latter’s addition to adsorption sites and desorption of K^+^ by co-application of PGPRs and biochar. Another PGPR *Burkholderia phytofirmans*, which is capable of producing exopolysaccharides, when inoculated along with biochar significantly, decreased salinity stress for plants by lowering Na^+^ content in soil solution. In addition to lowering Na^+^ content, co-applying PGPRs with biochar enhances colonization efficiency of the former thereby leading to synergistic effects on soil quality. For instance, Akhtar et al. [80] reported an increase in colonizing efficiency of PGPRs *Burkholderia phytofirmans and Enterobacter* sp. strains co-applied with 5% biochar (derived from hard wood and soft wood) in a saline soil than PGPRs without biochar in soil. *Enterobacter* sp with 5% biochar showed high colonizing efficiency in saline soil than *Burkholderia phytofirmans* with and without 5% biochar. Similarly, co-application of an endophytic PGPR with biochar to *Chenopodium quinoa* grown in a saline soil induced an increase of  ~ 150–250% in PGPR colonization in rhizosphere, root interior and shoot interior bacterial population than PGPR inoculation alone. In presence of biochar studies showed a decreased Na^+^/K^+^ ratio in soil and increased root colonizing efficiency of PGPRs hence alleviating salinity stress in soil. In soil solution, biochar and PGPRs maintain the nutrient balance by releasing mineral nutrients such K^+^, Ca^2+^ and Mg^2+^, thereby reducing Na^+^ in soil. This ultimately increased the K^+^/Na^+^ ratio in soil. Exopolysaccharide produced from PGPRs under stress binds Na^+^ in soil [80].

The use of PGPR in combination with biochar has also been studied in polluted soils (Table 2). On the basis of results, it emerges as a promising tool for reducing heavy metal contamination in the soil. For instance, Sabir et al. [16] found that *Enterobacter* sp. (PGPR) inoculums with biochar (paper and pulp derived) could be an efficient approach to accelerate remediation of soil contaminated with cadmium (Cd) (80 mg kg^−1^ soil). Although PGPR and biochar immobilized Cd in soil thereby mitigating its availability by 15.2% and 28.3%, respectively, their combination decreased it by 45.6%. Another PGPR, *Bacillus* sp. in the presence of biochar increased soil enzyme (dehydrogenase) 4.61 times high than biochar leading to increased bioremediation. This combination also decreased HOAc-extractable Cd level by 11.34% than sole applications of biochar or PGPR [70]. The application of *Bacillus* sp with 1% biochar significantly reduced the toxic effect of chromium and improved plant health by limiting the availability of the heavy metal [71]. Both PGPRs and biochar immobilizes metals through metal immobilizing bacteria, adsorption, co-precipitation, and complexation, thus reducing their availability in soil for uptake [103].

### Effect of co-application of PGPR and biochar on agriculture productivity under different stressors

Many studies have reported the effect of combined application of PGPR and biochar on plant productivity under different environmental stressors (Table 2). They have studied and invoked various physiological attributes to explain the effect of combined application of PGPR and biochar on plant growth and productivity. For instance, one of the effects of drought stresses is increase in ethylene levels in plants. It has been shown that the drought-induced increased ethylene level in plants can be mitigated by using ACC deaminase producing PGPR in conjunction with biochar because the latter supports the survival rate of inoculants and increases colonization in the plant rhizosphere [73]. This led to increased plant yields as compared to only PGPR or biochar application. Similarly, it was found in another study that co-applying ACC deaminase producing PGPRs *Achromobacter xylosoxidans*, *Pseudomonas aeruginosa*, *Leclercia adecarboxylata*, and *Enterobacter cloacae* with timber waste biochar (0.75 and 1.50% w/w) in drought conditions improved the growth of maize by inducing higher nutrients uptake and lower ethylene level than sole application of biochar or PGPR [104]. Briefly, they reported that *A. xylosoxidans* + 1.50% biochar showed 19 and 6% higher transpiration rate, 30 and 7% higher photosynthetic rate, and 16% and 7% higher stomatal conductance, respectively, than alone *A. xylosoxidans* or 1.5% biochar under severe drought. *E cloacae* + 1.5% biochar increased chlorophyll a by 26 and 13%, carotenoids by 28 and 4%, and total chlorophyll by 29 and 9%, respectively, than *E. cloacae* or 1.5% biochar, respectively. Similarly combined application of *P. aeruginosa* and biochar decreased electrolyte leakage by 28% and 4% than applying *P. aeruginosa* or biochar alone, respectively. Similarly, Nafees et al. [65] investigated combined use of *Cellulomonas pakistanensis* and *Sphingobacterium pakistanensis* PGPRs and biochar derived from wood of *Morus alba* (5% w/w) on *Vicia faba* under drought stress. They found that co-application positively ameliorated fresh and dry leaf weight by 28.57 and 10.47%, fresh and dry root weight by 36.36 and 14.28%, and fresh and dry shoot weight by 16 and 10% than sole application of biochar or PGPR, respectively. Some other ACC deaminase producing PGPRs, i.e., *Agrobacterium fabrum* and *Bacillus amyloliquefaciens* have also been found to boost wheat productivity under severe drought when used in combination with timber waste biochar [73]. *B. amyloliquefaciens* + biochar increased plant height by 34 and 24%, root length by 25 and 8%, and spike length by 5 and 2% than *B. amyloliquefaciens* or biochar alone. Similarly, *A. fabrum* + biochar increased 1000-grains weight by 13% when compared to sole application of *A. fabrum*. Ullah et al. [76] evaluated the effect of co-application of a PGPR, *Serratia odorifera,* and algal biochar on maize growth under drought stress. The co-application increased maize growth parameters like plant height by 38 and 16%, shoot fresh weight by 29 and 17%, shoot dry weight by 44 and 24%, root fresh weight by 60 and 27%, root dry weight by 84% and 24%, and root length by 47 and 32% than sole application of PGPR or biochar under severe drought stress, respectively. Decreased proline content due to combined application of PGPRs and biochar has also been cited as drought alleviating mechanism [60]. The PGPRs namely *Pseudomonas koreensis* and *Bacillus coagulans,* when used with biochars, on rice plant under drought conditions increased relative water content, stomatal conductance, Ca^2+^ and K^+^ content and decreased proline content in plants. Another PGPR, *P. fluorescens,* when applied along with biochar to cucumber under limited moisture conditions was found in much higher number than when it was applied alone [59]. Their combined application under severely limited moisture conditions improved shoot length, shoot fresh weight, root length, and root fresh weight by 10%, 10%, 29% and 16%, respectively, than the sole application of biochar. Also in PGPR + biochar treatment chlorophyll content and relative water content increased by 5% and 6% than biochar only treatment. They also found reduced electrolyte leakage which helped plants to deal with water stress conditions. Drought elevates ethylene and electrolyte leakage in plants leading to retardation of plant growth. Overall, co-application of PGPR with biochar can alleviate drought stress in plants by lowering ethylene content and electrolyte leakage in plants. PGPR with biochar found to increase to relative water content, stomatal conductance, chlorophyll, carotenoids in plants.

Soil salinity affects plant growth, development and photosynthesis. It also affects protein synthesis and lipid metabolism [105]. Plant growth under saline soils is adversely affected by osmotic effects and hormonal imbalances. It also causes malnutrition and specific ion toxicity [106]. Other reason is growth is inhibited by sodium and chloride ions as sodium ions are retained in roots and stems and in some plants only chloride ions are concentrated in the shoot which has a negative effect on plants [107, 108]. Co-application of PGPRs and biochar usually exerts synergistic effects on alleviating salinity stress and increasing plant productivity than their individual effects. For example, a siderophore-producing strain, *Burkholderia phytofirmans* in combination with tree-twig derived biochar improved plant height, root dry weight, shoot dry weight, grain yield, photosynthetic rate, stomatal conductance of *Chenopodium quinoa* by 17, 26, 10, 5, 5, 16 and 12%, respectively, under saline conditions than individual PGPR application only [68]. Evidence from multi-year field studies has also confirmed the synergistic potential of combining PGPR and biochar to alleviate soil salinity stress for plants. For instance, PGPR strains *Bacillus coagulans* and *Pseudomonas koreensis* were co-applied with rice husk-derived and corn stalk-derived biochars in a rice field having electrical conductivity of 4.67 dS m^−1^ biochar. The co-application alleviated the negative effects of salinity by decreasing Na^+^ content by 15.34% and 15.73%, and proline content by 52.49% and 49.57% in first and second year of the study, respectively, in rice leaves, in comparison to the uninoculated control [60]. Similarly, Akhtar et al. [80] found 25% and 8% less Na^+^ uptake than biochar or PGPR sole applications, respectively, by using *Enterobacter* with 5% biochar *and Burkholderia phytofirmans* with 5% biochar derived from hard and soft wood in saline soil.

PGPR and biochar play an important role in the management of heavy metal stress in plants. They can transform, accumulate or detoxify heavy metals [109]. For instance, Zafar-ul-Hye et al. [66] found 13.5% less uptake of Pb in mint leaves after it was inoculated with ACC-deaminase producing PGPRs, *Alcaligenes faecalis* and *Bacillus amyloliquefaciens* and provided with compost (mixed fruits) mixed biochar (vegetable waste). Resultantly, they found that *A. faecalis* strain along with compost-mixed biochar significantly improved plant chlorophyll content by 37%, root dry weight by 58%), nitrogen by 46%, phosphorus by 39%, and potassium by 63% in mint leaves than untreated control. In another study, the lead uptake in spinach decreased by 43% whereas potassium uptake increased by 10.5% over untreated control by the use of compost-mixed biochar and *Bacillus amyloliquefaciens* strain [69]. The PGPR *Enterobacter* sp. when co-applied along with biochar significantly enhanced growth of *Brassica napus* in cadmium-spiked (80 mg kg^−1^) soil [16]. The co-application significantly increased shoot and root length by 52.5 and 76.5%, respectively, than sole application of PGPR, by 22 and 34.8% than soil without PGPR and by 29 and 41.6% sole application of biochar under stress. PGPR + biochar treatment also decreased Cd uptake by 40.1 and 38.2% in root and shoot than PGPR (16.8 and 16.9%), and biochar (23.4 and 21.3%), respectively, as compared to control under Cd stress conditions. Ma et al. [70] found an increase in ryegrass biomass (1.96 g pot^−1^) than biochar only (0.42 g pot^−1^) and lowest Cd concentration (5.45 mg kg^−1^) was found in PGPR + biochar treatment as compared to biochar, PGPR and control (soil without PGPR and biochar).

## Mechanistic understanding of interaction of PGPR and biochar

The synthesis of literature so far in this paper has amply highlighted that the biochar and PGPRs work synergistically in improving the soil quality and agriculture productivity. When biochar is applied with PGPR inoculants, it provides habitat for PGPR (i.e., colonization, reproduction and growth) due to its porous structure and high surface area and also the ability to adsorb microorganisms and organic compounds [110]. Some studies cited in the previous sections have suggested this by showing higher growth and abundance of PGPR inoculants when biochar is also applied to soils. Biochar also protects them from other harmful pathogens [111]. Owing to richness in carbon, i.e., substrate, and essential nutrients, it provides both energy and the required nutritive building blocks for inoculants’ survival and growth [112]. In addition, biochar modifies physicochemical properties of soils that may lead to increase in soil microbial biomass and enzymatic activity [29, 98]. Biochar is rich in a range of mineral nutrients including nitrogen, phosphorus, potassium, calcium, magnesium, zinc, etc., depending upon the feedstock type and pyrolysis temperature [113]. Upon addition to soil, it is decomposed gradually to release these nutrients in the soil solution [114–116].

PGPRs are involved in plant growth promotion under normal and stressed conditions through their direct and indirect mechanisms. Similar to biochar, the PGPR may either bring in a nutrient from outside through their direct mechanism such as nitrogen fixation (by nitrogen-fixing bacteria) or solubilize the immobilized nutrients (by phosphate-solubilizing bacteria) thereby contributing to plant nutrition. For instance, nitrogen-fixing PGPRs such as *Paenibacillus polymyxa*, *Rahnella* sp., *Serratia* sp. have the ability to enhance the mineral nitrogen content in soil solution through their nitrogen-fixing traits and prevents its leaching in soil [56, 117]. A large number of phosphate-solubilizing PGPRs, e.g., *Bacillus* sp.,* Bacillus lentus*, *B. subtilis*, *Bacillus megaterium*, *Burkholderia* sp., *Glomus etunicatum*, *G. mosseae*, *Pseudomonas* species, *Pseudomonas fluorescencs Penicillium* strains, *Lysinibacillus fusiformis*, *Azotobacter chroococcum*, *Azospirillum brasilense*, *Arthrobacter*, *Streptomyces*, have been shown to solubilize and provide phosphate in soil for plant uptake [40, 49, 51, 53, 56, 77, 91, 112, 117, 118]. While the direct accrual of phosphorus from biochar by co-applied PGPR has not been demonstrated in any study, it can be safely speculated that such a mechanism exists. A similar mechanism of enhanced availability of potassium can be assumed because PGPR are known for lowering the soil pH and making the soil potassium available to plants and biochar are known to be rich in potassium [28, 96]. Another direct mechanism is production of ACC deaminase which lowers the production of ethylene elevated level produced under stress conditions through its breakdown into ammonia and alpha ketobutyrate [119]. PGPRs such as *Enterobacter* sp., *Alcaligenes* sp., *Pseudomonas fluorescens*, *Serratia odorifera*. *Leclercia adecarboxylata*, *Agrobacterium fabrum*, *Bacillus amyloliquefaciens*, *Pseudomonas aeruginosa*, etc., have the ability to produce ACC deaminase. These strains show synergistic effects with biochar in abiotic stress alleviation [59, 72, 73, 80, 104]. PGPRs through their indirect mechanisms such as pH regulations, production of exopolysaccharides, protection against plant diseases are also involved in plant growth promotions [120].

## Conclusions and perspective

Under different environmental stresses, low crop growth and crop failure is the norm across many important food and cash crops. Co-application of PGPR and biochar offers a sustainable, cost-effective, and environment-friendly technique for increasing crop productivity and improving soil quality. Even under normal conditions, this combination may act synergistically to improve crop productivity as well as soil quality in addition to lowering the need for chemical fertilizers. However, as is highlighted by this review, there are not many field experiments that have been conducted to explore the potential of combined application of the PGPR and biochar for sustainable food production. Given the state-of-the-art of the subject, we have following recommendations for future studies:

- Mechanistic understanding of the interaction between PGPR and biochar needs further exploration. For instance, currently we don’t know exactly if the synergistic effect of the two is because of the conducive habitat afforded to the PGPR by biochar or it is due to the enhanced availability of substrate and nutrients due to biochar that sustains and promotes the PGPR. It can be done by using isotopically labeled biochar (i.e., ^13^C, ^15^ N, ^33^P) in order to trace the carbon and nutrients accrued into microbial biomass. Concurrently, the colonization efficiency of the PGPR should also been estimated.
- Long-term field experiments could be a highly effective way of evaluating the combined effect of the PGPR and biochar. Individually, the PGPR and biochar have been assessed in reasonably long-term experiments for their potential for sustainable food production [12–14, 121]. However, they should now be assessed together in multi-year field experiments under the assumption that the biochar keeps influencing soil properties with aging, whereas the PGPR might persist longer in biochar-amended soils.
- The PGPR technology is not very successful in degraded soils situated in semi-arid and arid areas, especially which are poor in soil organic matter, because the PGPR have not good reserves of substrate and nutrient-source for their growth and function. Combined application of the PGPR and biochar in these soils could be a very good strategy and needs to be assessed. The biochar may provide the PGPR the habitat to survive and flourish as well as the necessary substrates, which are lacking in such soils, and is the key reason of failure of PGPR technology there.
- Meta-analyses of the studies on biochar vis-à-vis agricultural productivity have revealed that the major mechanism by which they improve productivity is the liming effect [19, 82]. Such biochars, when combined with phosphate-solubilizing bacteria that prefer acidic or near-neutral pH will not give good results. Therefore, the studies should combine the biochar and PGPR after keeping into account such complementarities.

## Data Availability

Not applicable.

## Abbreviations

PGPR: Plant growth-promoting rhizobacteria
BC: Biochar
NUE: Nitrogen use efficiency
SOC: Soil organic carbon
IAA: Indole acetic acid
NaCl: Sodium chloride
FC: Field capacity
N: Nitrogen
P: Phosphorus
K: Potassium
Pb: Lead
Cd: Cadmium
